# Sternoclavicular Joint Septic Arthritis and Anterior Mediastinal Mass in a Young Athlete: Possible Immune-modulatory Effect of Growth Hormone

**DOI:** 10.7759/cureus.6155

**Published:** 2019-11-14

**Authors:** Tahir Muhammad Abdullah Khan, Abdul Hasan Siddiqui, Yusra Ansari, Saad Ali Ansari, Faraz Siddiqui

**Affiliations:** 1 Internal Medicine, Marshfield Medical Center, Marshfield, USA; 2 Pulmonary and Critical Care Medicine, Staten Island University Hospital / Northwell Health, Staten Island, USA; 3 Internal Medicine, Rawalpindi Medical College, Rawalpindi, PAK; 4 Internal Medicine, Pakistan Institute of Medical Sciences, Islamabad, PAK; 5 Pulmonary and Critical Care Medicine, Robert Packer Hospital, Sayre, USA

**Keywords:** growth hormone, mediastinal mass, sternoclavicular joint, septic arthritis, methicillin sensitive staphylococcus aureus, drug abuse, immune modulation

## Abstract

Septic arthritis of sternoclavicular joint (SCJ) is a rare disease, however, not uncommon in patients who abuse intravenous drugs. It can present with a wide range of manifestations that can pose diagnostic challenges, which can result in a delay in diagnosis and treatment. Over the last few decades, there is a surge in the use of nonprescription recombinant human growth hormone (rhGH) by the young healthy population and athletes for its purported ergogenic effects. Furthermore, we lack quantitative information about the adverse effects of the chronic use of rhGH in a healthy population due to the scarcity of epidemiological data. We are reporting a case of a young male athlete who was chronically using the subcutaneous rhGH formulation to build lean body mass, and presented with septic arthritis of right SCJ due to methicillin-sensitive Staphylococcus aureus (MSSA) complicated by a necrotic inflammatory response involving the mediastinum which infiltrated the apical lung parenchyma. The clinical presentation masqueraded as the mediastinal mass raising the suspicion of mediastinal malignancy. Histological analysis of the tissue of SCJ and mediastinal area revealed no malignant cells but a lymphocyte-predominant inflammatory response with germinal centers was observed, which was an atypical response to MSSA bacterial infection. We have reviewed the literature to elucidate the immune-modulatory effect of rhGH, as the chronic use of rhGH by our patient probably has contributed to an atypical immune response to MSSA. The patient was treated with an extended duration of parenteral antibiotics and multiple incision and debridements to achieve complete resolution of infection over the next six months. This is a unique case of septic arthritis of right SCJ in a patient on chronic subcutaneous rhGH which masqueraded as a mediastinal mass raising concern of malignancy; moreover, it highlights the probable immune-modulatory role of rhGH which instigated an atypical immune response to MSSA infection.

## Introduction

According to a survey conducted to estimate the prevalence of use of ergonomic aids among the athletes, 5% of recreational athletes reported the use of growth hormone (GH) intending to increase the lean body mass and strength [1]. The use of ergogenic drugs has doubled over the last decade [1], and this high prevalence is attributed to its easy availability in the market [1]. Although pathophysiological and metabolic effects of recombinant human growth hormone (rhGH) are well known, the immune-modulatory effects of rhGH are not well studied. We report a case of septic arthritis of the sternoclavicular joint (SCJ) in a patient using nonprescription rhGH supplement, who presented with an anterior mediastinal mass mimicking a mediastinal neoplastic process. Unusual presentation of the disease can pose a diagnostic challenge and as a result can lead to a delay in diagnosis and the initiation of treatment. Therefore, it is important to apprise the healthcare providers of the atypical presentation of septic arthritis of SCJ, and, also, it highlights the possible immune-modulatory effect of rhGH which can result in an altered and unexpected inflammatory response to various infectious organisms.

## Case presentation

A 33-year-old male athlete with no significant past medical history presented with gradually worsening right arm and right handgrip weakness which started three days prior to presentation. The patient reported a painless swelling on the right side of the chest wall slowly growing over the last three months, and it was associated with malaise, intermittent night sweats, anorexia, and an unintentional weight loss of 15 pounds over the same period of time. The patient denied having fever, chills, or any other focal neurological deficits. Social history was negative except for the use of subcutaneous rhGH on a daily basis over the last 10 years with an intent to build lean body mass. The patient was actively involved in endurance physical exercise training and heavy weight lifting. The patient denied any trauma to left shoulder or chest wall except hearing a “pop” in his right shoulder during weight lifting four months ago that led to an acute onset of transient mild pain in the right shoulder for which he was worked up to rule out any musculoskeletal injury with CT and MRI that were negative for any musculoskeletal injury. Subsequently, the patient underwent physical therapy for a month with improvement in pain.

On admission, physical exam revealed a well-developed and well-nourished man with an athletic built and a 2 cm x 3 cm nontender mass at the anterior superior part of the right chest wall involving the right SCJ and soft tissues of the neck. There was the demonstrable weakness in the flexion and abduction of the right shoulder with a weakness in the right handgrip. The rest of the examination was normal.

**Figure 1:**
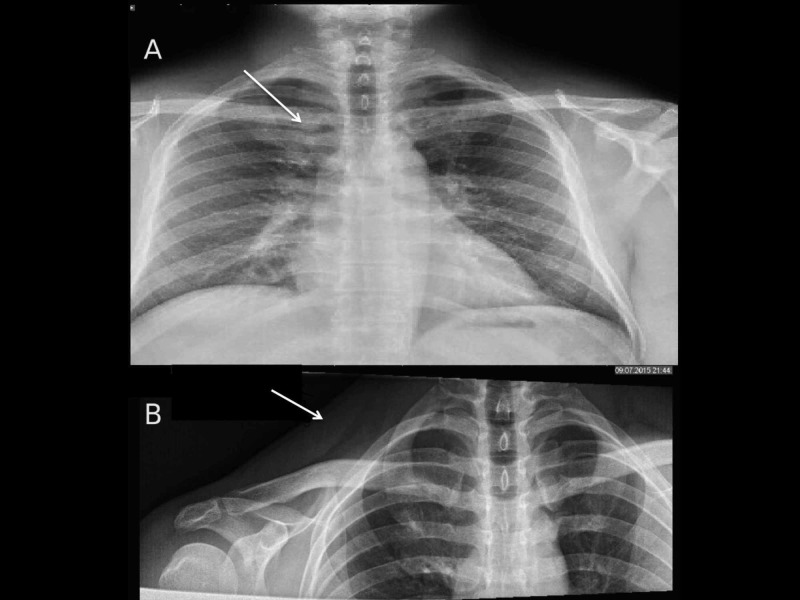
Chest X-ray AP view and right clavicle X-ray AP view. A: Chest X-ray AP view showing a dense opacity in the right para-tracheal area (arrow). B: Right clavicle X-ray AP view showing soft tissue inflammation on the right side of the base of the neck (arrow), but no evidence of fracture or dislocation of the clavicle bone. AP, anteroposterior

His laboratory studies were significant for an elevated erythrocyte sedimentation rate (ESR) and C-reactive protein (CRP) that were 122 mm/h and 6 mg/L, respectively. Blood cultures and oropharyngeal cultures were drawn on admission that came back negative. Initial imaging with chest X-ray revealed a right paratracheal haziness (see Figure 1A), and the right clavicle X-ray showed soft tissue inflammation of the base of the neck on the right side, but no fracture or dislocation of the clavicle was observed (see Figure 1B).

**Figure 2:**
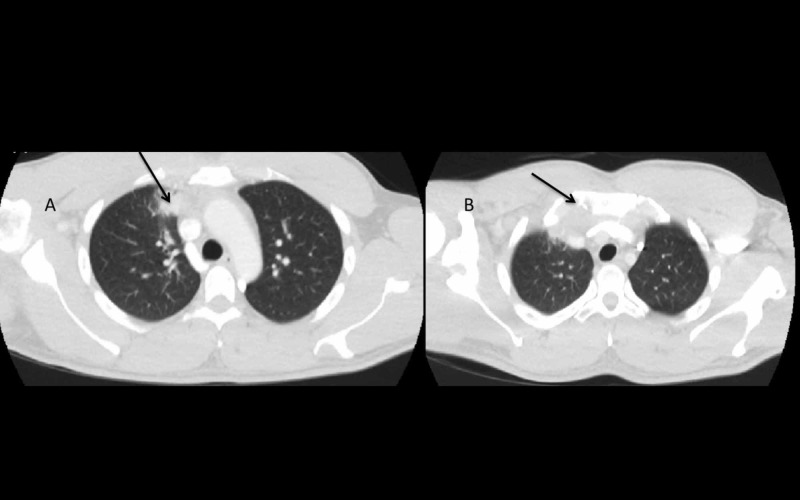
CT of the chest with contrast. A: CT chest with contrast showing focal 2.2 cm x 2.6 cm x 2.9 cm anterior-superior mediastinal mass with invasion into the right lung parenchyma (see arrow). B: CT chest with contrast showing focal anterior-superior mediastinal mass infiltrating the manubrium sterni (see arrow).

A growing mass on the right side of the chest wall with focal neurological deficit i.e., weakness of right arm abduction and weak right hand-grip, associated with X-ray findings suspicious of a malignant lesion in the paratracheal area, prompted a CT scan of the chest with contrast that revealed a 2.2 cm x 2.6 cm x 2.9 cm anterior mediastinal mass in the superior-medial aspect of mediastinum invading the lung and the pleural cavity, and it also involved the right SCJ and manubrium sterni (see Figure 2). Also, a significant hilar and mediastinal lymphadenopathy was noted suggestive of a highly aggressive inflammatory process due to either an advanced malignancy or an infectious process in the mediastinum.

**Figure 3:**
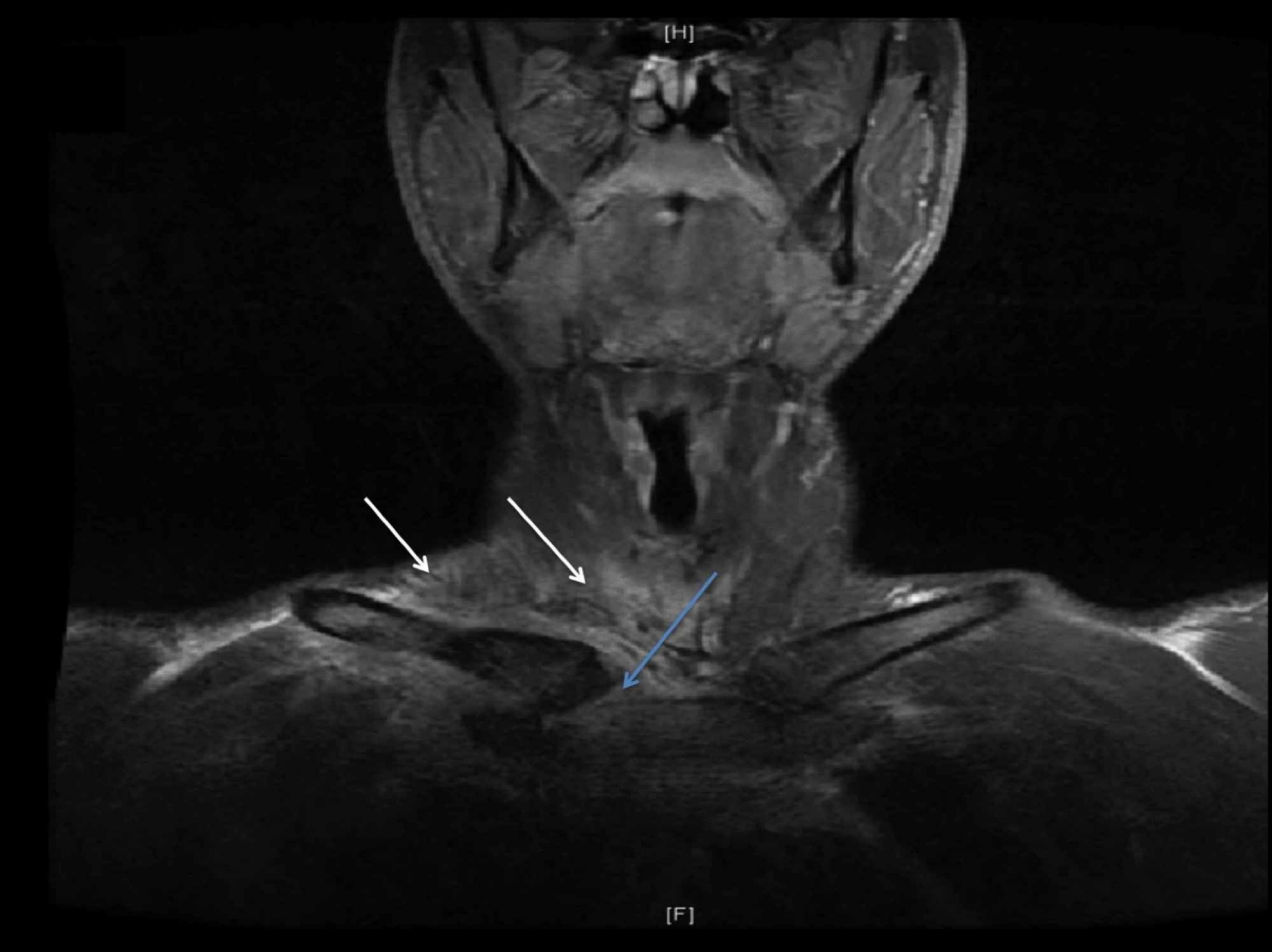
MRI of the neck and SCJ with contrast. MRI of the neck and SCJ with contrast showed large infiltrative process involving the right SCJ (blue arrow) and the surrounding soft tissues with extension into the base of the neck on the right side of the midline (white arrows). SCJ, sternoclavicular joint

Subsequently, MRI of the right neck and SCJ with (see Figure 3) and without contrast were performed that revealed inflammation of the right SCJ extending from the soft tissue at the base of the neck to the anterior superior mediastinum. No evidence of injury to the right brachial plexus was observed; however, the soft tissue around the brachial plexus was edematous due to the inflammation.

**Figure 4:**
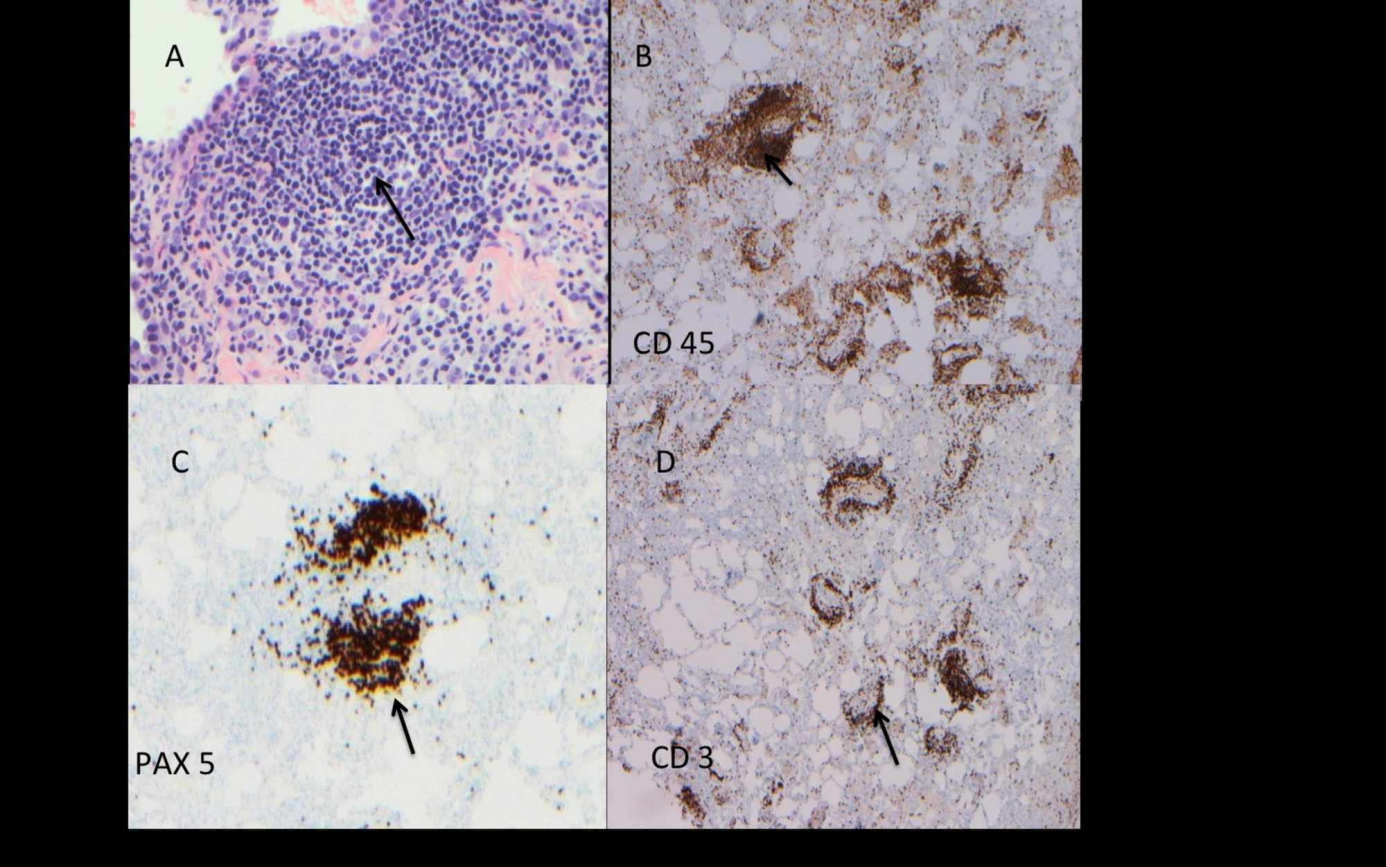
Histopathology of SCJ, mediastinal tissue, and lung parenchyma. A: At high magnification (40X), infiltrating diffuse lymphocytes are seen forming the germinal center (arrow). B, C: The histology slides showing inflammatory infiltrate comprised of a mixture of CD 45 and PAX 5 positive B lymphocytes (arrow). D: The histology slide showing CD 3 positive T lymphocytes (arrow). SCJ, sternoclavicular joint

To further elucidate the etiology of inflammation observed on imaging studies, a CT scan guided fine needle aspiration (FNA) biopsy of the mass was performed which showed chronic inflammatory cells with a predominance of B and T lymphocytes, macrophages, and histiocytes with a few atypical cells. Furthermore, Gram stain, cultures, and acid-fast bacilli (AFB) smear of tissue biopsy were negative. Considering a high suspicion for malignancy i.e., lymphoma or teratoma, and low sensitivity of CT-guided FNA biopsy to diagnose mediastinal tumors, cardiothoracic surgery was consulted for mediastinoscopy with tissue biopsy. During the mediastinoscopy, an aggressive inflammatory process in the right anterior mediastinum was observed that invaded the right SCJ in cephalad direction, and posteriorly and caudally, it progressed to involve the right apical lung parenchyma. The patient underwent incision and debridement of the necrotic mediastinal tissue with a scraping of SCJ, and the tissue was sent for histology and culture which confirmed the diagnosis of complicated septic arthritis of SCJ. The histological analysis demonstrated the formation of germinal centers in the lung parenchyma and a predominance of CD 3 positive T lymphocytes, CD 45, and PAX 5 positive B cell lineage lymphocytes were observed in addition to macrophages, histiocytes, and dendritic cells (see Figure 4 A-D). The tissue cultures grew methicillin-sensitive *Staphylococcus aureus* (MSSA) establishing an infectious process.

**Figure 5:**
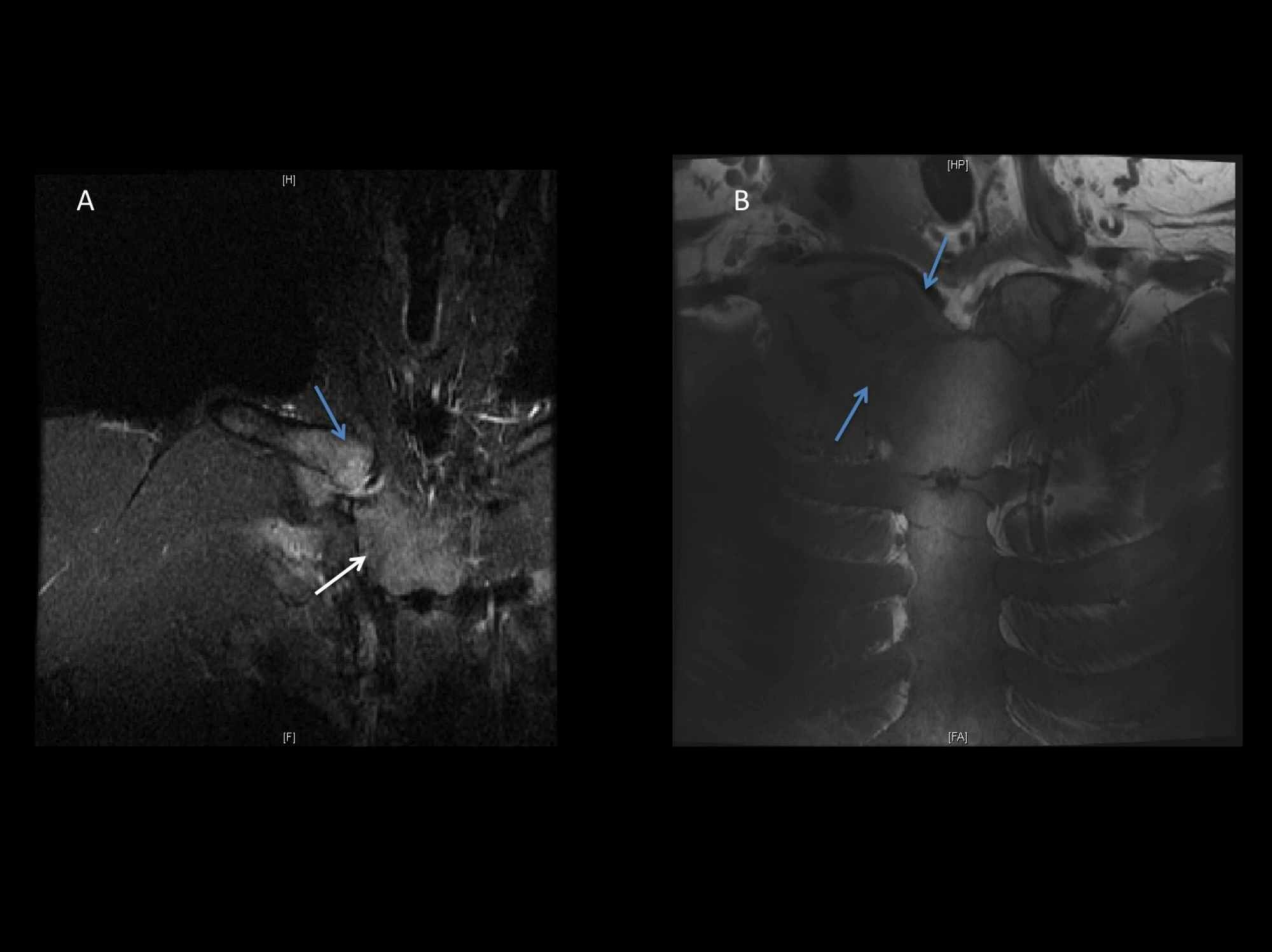
Follow-up MRI of the chest and right SCJ with and without contrast. A: MRI chest and right SCJ with contrast showing right sternoclavicular septic arthritis (blue arrow) with manubrium osteomyelitis (white arrow). B: MRI chest and right SCJ without contrast showing inflammatory changes around the right SCJ. SCJ, sternoclavicular joint

The patient was started on intravenous clindamycin and nafcillin during hospitalization, and later on, patient was discharged home on long-term intravenous antibiotics with nafcillin. The patient was advised to abstain from the use of any GH products, and avoid heavy weight lifting till complete recovery. At six weeks follow up, due to inadequate response to antibiotics, a follow-up MRI of the chest with and without contrast revealed an active inflammation of SCJ and signs of osteomyelitis of manubrium (Figure 5A-B). A repeat mediastinoscopy with incision and debridement of the SCJ tissue and the lung pleura was performed and an extended parenteral antibiotic course was recommended. 

 

**Figure 6:**
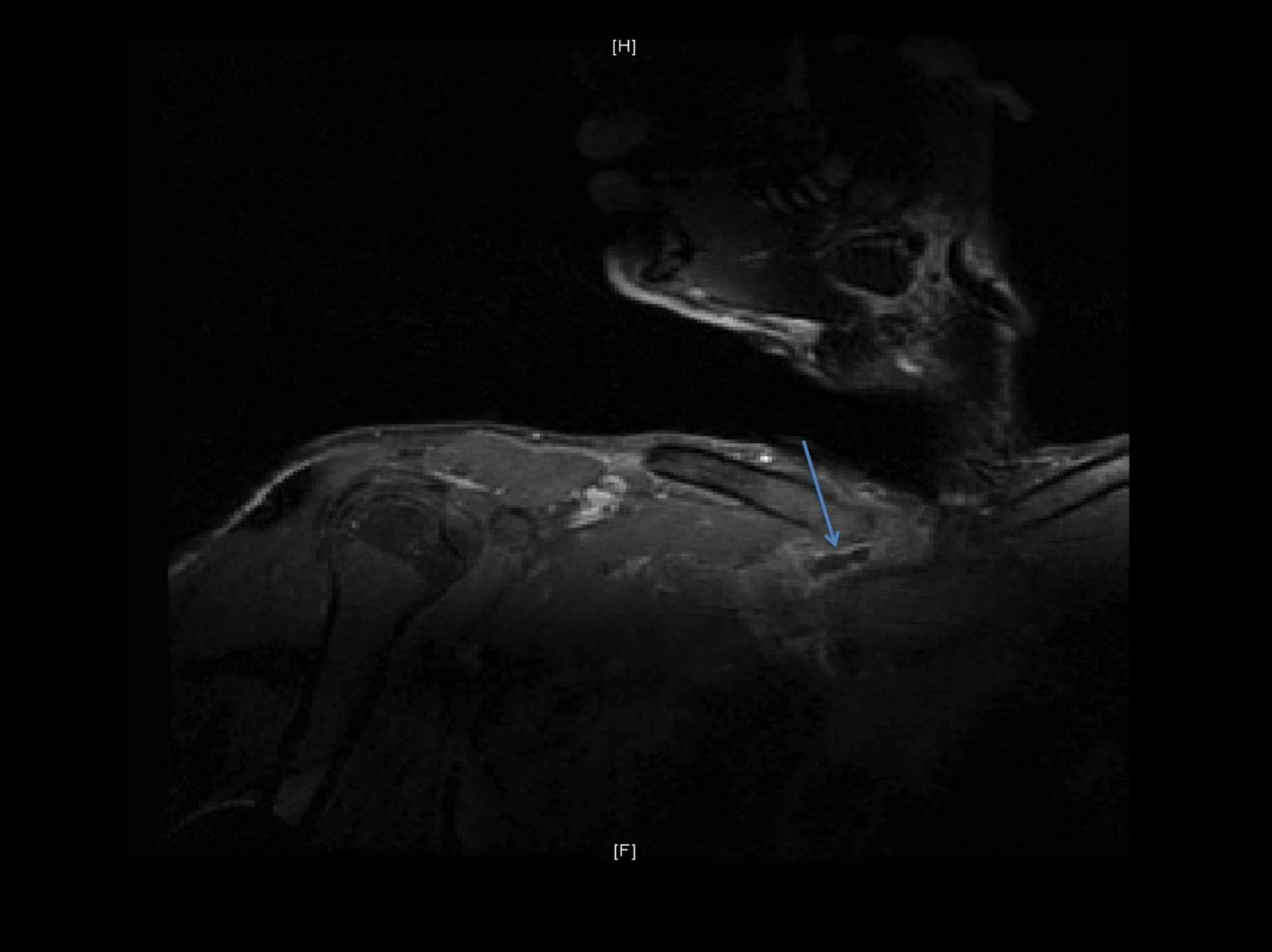
MRI of the right SCJ with contrast. Follow-up MRI of the right SCJ with contrast demonstrated old postsurgical changes in the SCJ and surrounding tissues but no significant contrast enhancement is visualized which endorsed the resolution of the infectious process. SCJ, sternoclavicular joint

At six months follow-up, the patient showed complete recovery with a resolution of SCJ septic arthritis and it was confirmed by the follow-up MRI of right SCJ with and without contrast (see Figure 6).

## Discussion

The rhGH is one of the many performance-enhancing drugs prevalent among both the professional as well as nonprofessional athletes to build lean body mass and enhance the exercise capacity to improve sporting performance [1]. The evidence about high prevalence of rhGH abuse among the young population came from the anecdotal reports and the surveys of recreational athletes available in the literature [1]. There are varieties of GH products abused by the athletes for ergogenic effects that include: 1. rhGH, which acts on GH receptors. 2. GH secretagogues, i.e., GH releasing hormone (GHRH) and its analogs; GH releasing peptides (also known as ghrelin analogs) which acts on ghrelin receptors to enhance GH release; and the amino acids, e.g., ornithine or arginine, to enhance the secretion of GH [2-3].

Illicit distribution and use of the rhGH usually stem from prescribing it for off-label uses or for the treatment of medical conditions without examination or supervision. Moreover, rhGH is readily available through Internet pharmacies, wellness and anti-aging clinics, and from the websites which market rhGH with other performance-enhancing drugs. Easy accessibility along with lack of awareness of side effects of rhGH may be a contributing factor to the overuse of GH supplement as seen in our patient [4].

The rhGH increases the lean body mass; however, its effect to enhance the strength or exercise capacity is still unproved [5-6]. In addition, the use of rhGH is not devoid of adverse effects. Although there are no formal epidemiological studies available to describe the adverse impact of the use of rhGH in healthy young athletes, its detrimental effect is usually derived from the clinical and observational studies of rhGH supplementation in adult patients with hypopituitarism. Edema is reported as the most common side effect due to anti-natriuretic effect of GH with increased retention of renal sodium and water retention. Moreover, these patients can experience arthralgia, myalgia, carpal tunnel syndrome, paresthesia, and decreased insulin sensitivity [6].

We have reported a case of young athletic male abusing subcutaneous rhGH who presented with complicated SCJ septic arthritis secondary to MSSA infection with an insidious clinical course. Importantly, we assume that rhGH may have contributed to atypical presentation of septic arthritis due to its immunomodulatory properties. SCJ septic arthritis accounts for 1% of all septic arthritis in the general population with incidence rising to 17% among patients abusing intravenous drugs [7]. Mean age of the patients with SCJ infection is reported as 45 years with 73% being male [7]. SCJ septic arthritis has a wide range of clinical presentations, i.e., chest pain, the most common initial symptom (78%), followed by the shoulder pain (24%) [7], and it can also manifest with a wide range of complications, i.e., osteomyelitis, chest wall abscess or phlegmon, and/or mediastinitis [7]. However, the presentation of septic arthritis of SCJ as an anterior mediastinal mass infiltrating the apical lung parenchyma and the brachial plexus leading to right arm weakness, as seen in our patient, has not been reported previously in the literature. The common risk factors for SCJ septic arthritis are intravenous drug abuse (21%), distant skin infection (15%), diabetes mellitus (13%), trauma to clavicle bone or joint (12%), end-stage renal disease, chronic liver disease, alcohol abuse, immunodeficiency states, infected central venous lines, and rheumatoid arthritis [7]. However, in a case series of 180 patients with SCJ infection reported by Ross et al., for a significant number of patients with SCJ septic arthritis (23%) no specific risk factor was identified [7]. *Staphylococcus aureus* is the most common causative organism in both healthy individuals as well as intravenous drug abusers [7]. Previously, *Pseudomonas aeruginosa* was the most common causative organism in intravenous drug abusers [8]; however, after the end of epidemic of pentazocine abuse the incidence of *P. aeruginosa* infection has declined [7]. Other causative organisms reported in the literature are: *Escherichia coli*, *S. pneumonia*, *Mycobacterium tuberculosis*, *Pasteurella multocida*, *group B Streptococcus*, *Mycobacterium avium* intracellular, *Fusobacterium necrophorum*, and *Bacteroides oralis* [9].

Although our patient did not use intravenous drugs, the use of subcutaneous injections of rhGH probably had caused the transient MSSA bacteremia with seeding of SCJ. This pathogenic mechanism is consistent with a previously reported case of SCJ septic arthritis due to transient MSSA bacteremia caused by acupuncture therapy [10].

A wide array of presentations of SCJ infection and a possible insidious course of illness without a systemic inflammatory response may lead to a delay in diagnosis and treatment; therefore, a high index of suspicion should be kept to timely diagnose and treat the SCJ infection. Furthermore, negative blood cultures or having no obvious source of infection should not preclude SCJ infection from the differential diagnosis. In the case series by Ross et al. only 62% of patients with SCJ septic arthritis had bacteremia [7]. Moreover, imaging studies with CT or, preferentially, MRI of the SCJ should be obtained promptly to confirm a diagnosis and to identify the complications warranting surgical treatment. The treatment of choice for septic arthritis of SCJ is parenteral antibiotic therapy with or without surgical intervention [7, 11-12].

In our patient, imaging studies of SCJ and chest showed SCJ infection complicated by phlegmon in the mediastinum that mimicked anterior mediastinal mass. Subsequently, histopathological analysis of tissue of the SCJ revealed lymphocyte and monocytes predominant inflammatory response without any evidence of malignant cells. Later on, the culture of the SCJ tissue grew MSSA which confirmed the infectious process. It is important to note that the infections secondary to bacterial infection with MSSA usually instigate neutrophils predominant response. However, in our case, the infection of SCJ with MSSA precipitated a lymphocyte and monocyte predominant inflammation. We assume that the chronic use of rhGH may probably have played an immunomodulatory role that created an atypical inflammatory response to the MSSA bacterial infection. In 1976, Lesniak and Roth elucidated the immunomodulatory effect of GH and demonstrated the growth hormone receptor (GHR) on the immune cells [13]. Subsequently, Badolato et al. validated the expression of GHR on the lymphocytes who demonstrated a strong expression of GHR observed on CD20+ B lymphocytes, and a comparatively weaker expression of GHR was observed on CD 2+ Lymphocytes i.e., T lymphocytes and natural killer cells (NK) [14]. Dendritic cells (DC) are key effectors for T cell and B cells, and result in generation of immunogenicity. Human growth hormone (hGH) induces an increased expression of co-stimulation markers on DCs, and also augment the chemokines and cytokines i.e., interleukin-6, interleukin-8, IL 12P40, CCL 2, CCL 3, CCL4, and CXCL-10 [14-15]. As a result, the activated modified DC leads to polarization of Helper T cells (Th1) resulting in enhanced proliferation of allogenic T cells and cytokines production [15]. Moreover, GH also enhances thymopoiesis resulting in increased production of T lymphocytes [16]. In addition to its effect on T cells, the GH suppresses the differentiation of B-lymphocytes into plasma cells, and it hampers the chemotactic response of plymorphonucleocytes (PMN) and causes a sluggish chemotaxis of PMN to the infection site [17-18]. But, on the other hand, GH enhances the chemotaxis of monocytes in a dose-dependent fashion towards the infection site [18-19], thus, leading to a chronic inflammatory response as seen in our patient. On histopathological examination of the tissue obtained from SCJ of our patient, an inflammatory response with predominance of T lymphocyte adjunct to paucity of PMN (Figure 3A-D) correlates with immunomodulatory effect of GH as described in the literature. In summary, the sluggish chemotaxis of neutrophils with reduced differentiation of B-lymphocytes into plasma cell, and a modified aggressive response of T lymphocytes in adjunct to enhanced chemotaxis of monocytes to the SCJ infection site may have contributed to an insidious manifestation of SCJ infection which mimicked an anterior mediastinal mass on initial presentation.

## Conclusions

We presented a case of an atypical presentation of septic arthritis of SCJ in an athletic young patient who was using rhGH to enhance physical performance and to build the lean body mass. The rhGH possibly has an immunomodulatory role and that may have resulted in the atypical presentation of SCJ septic arthritis as seen in our patient. It is important to further elucidate the immune modulatory effect of rhGH and its clinical implications. In addition, the athletes and healthy individuals who intend to use rhGH should be made aware of the potential adverse effects of rhGH including its possible adverse impact on the immune system which can result in variable response to the infectious organism.

## References

[1] Raschka C, Chmiel C, Preiss R, Boos C (2013). [Recreational athletes and doping--a survey in 11 gyms in the area of Frankfurt/Main]. MMW Fortschr Med.

[2] Baumann GP (2012). Growth hormone doping in sports: a critical review of use and detection strategies. Endocr Rev.

[3] Chromiak JA, Antonio J (2002). Use of amino acids as growth hormone-releasing agents by athletes. Nutrition.

[4] (2018). Human Growth Hormone - DEA Diversion Control Division. https://www.deadiversion.usdoj.gov›drug_chem_info›hgh.

[5] Saugy M, Robinson N, Saudan C, Baume N, Avois L, Mangin P (2006). Human growth hormone doping in sport. Br J Sports Med.

[6] Liu H, Bravata DM, Olkin I (2008). Systematic review: the effects of growth hormone on athletic performance. Ann Intern Med.

[7] Ross JJ, Shamsuddin H (2004). Sternoclavicular septic arthritis: review of 180 cases. Medicine.

[8] Bayer AS, Chow AW, Louie JS, Guze LB (1977). Sternoarticualr pyoarthrosis due to Gram-negative bacilli. Report of eight cases. Arch Intern Med.

[9] Youssef D, Bhargava A (2019). Escherichia coli bacteremia with secondary seeding in the sternoclavicular joint: a case report and literature review. Germs.

[10] Liu B-M, Wang T-L, Hung S (2015). Sternoclavicular septic arthritis caused by acupuncture over the posterior neck. Eur J Case Rep Intern Med.

[11] Nusselt T, Klinger HM, Freche S, Schultz W, Baums MH (2011). Surgical management of sternoclavicular septic arthritis. Arch Orthop Trauma Surg.

[12] Rodchuae M, Ruangpin C, Katchamart W (2017). Clinical manifestations, treatment outcomes, and risk factors for sternoclavicular septic arthritis. Rheumatol Int.

[13] Lesniak MA, Roth J (1976). Regulation of receptor concentration by homologous hormone. Effect of human growth hormone on its receptor in IM-9 lymphocytes. J Biol Chem.

[14] Badolato R, Bond HM, Valerio G (1994). Differential expression of surface membrane growth hormone receptor on human peripheral blood lymphocytes detected by dual fluorochrome flow cytometry. J Clin Endocrinol Metab.

[15] Gallais Y, Szely N, Legrand FX, Leroy A, Pallardy M, Turbica I (2017). Effect of growth hormone and IgG aggregates on dendritic cells activation and T-cells polarization. Immunol Cell Biol.

[16] Tesselaar K, Miedema F (2008). Growth hormone resurrects adult human thymus during HIV-1 infection. J Clin Invest.

[17] Fornari MC, Palacios MF, Diez RA, Intebi AD (1994). Decreased chemotaxis of neutrophils in acromegaly and hyperprolactinemia. Eur J Endocrinol.

[18] Fornari MC, Scolnik MP, Palacios MF, Intebi AD, Diez RA (1994). Growth hormone inhibits normal B-cell differentiation and neutrophils' chemotaxis in vitro. Int J Immunopharmacol.

[19] Wiedermann CJ, Reinisch N, Braunsteiner H (1993). Stimulation of monocyte chemotaxis by human growth hormone and its deactivation by somatostatin. Blood.

